# *Microvirga massiliensis* sp. nov., the human commensal with the largest genome

**DOI:** 10.1002/mbo3.329

**Published:** 2016-01-08

**Authors:** Aurélia Caputo, Jean‐Christophe Lagier, Saïd Azza, Catherine Robert, Donia Mouelhi, Pierre‐Edouard Fournier, Didier Raoult

**Affiliations:** ^1^ Unité de Recherche sur les Maladies Infectieuses et Tropicales ÉmergentesCNRSUMR 7278 – IRD 198Faculté de médecineAix‐Marseille Université27 Boulevard Jean Moulin13385Marseille Cedex 05France; ^2^ Special Infectious Agents UnitKing Fahd Medical Research CenterKing Abdulaziz UniversityJeddahSaudi Arabia

**Keywords:** Culturomics, large genome, *Microvirga massiliensis*, taxonogenomics

## Abstract

*Microvirga massiliensis* sp. nov. strain JC119^T^ is a bacteria isolated in Marseille from a stool sample collected in Senegal. The 16S rRNA (JF824802) of *M. massiliensis* JC119^T^ revealed 95% sequence identity with *Microvirga lotononidis* WSM3557^T^ (HM362432). This bacterium is aerobic, gram negative, catalase positive, and oxidase negative. The draft genome of *M. massiliensis* JC119^T^ comprises a 9,207,211‐bp‐long genome that is the largest bacterial genome of an isolate in humans. The genome exhibits a G+C content of 63.28% and contains 8685 protein‐coding genes and 77 RNA genes, including 21 rRNA genes. Here, we describe the features of *M. massiliensis* JC119^T^, together with the genome sequence information and its annotation.

## Introduction

Culturomics was developed in 2012 in order to extend the human gut repertoire. In the first study, by multiplying the number of culture conditions (212 different culture conditions) with rapid identification of the colonies using MALDI‐TOF, we were able to identify 340 different bacterial species including 31 new bacterial species (Lagier et al. 2012a,b,c,d). In addition, we demonstrated a complementarity with metagenomics (the usual gold standard for studying complex ecosystems) because only 15% of the species were concomitantly detected by culture and metagenomics applied to the same samples. We applied this strategy to diverse stool samples from healthy individuals from diverse geographic origins and from patients with diverse diseases, dramatically extending our knowledge of the cultured human gut repertoire (Lagier et al. 2015).

The current bacterial taxonomy to define species traditionally combines phenotypic and genotypic characteristics (Stackebrandt and Ebers 2006; Tindall et al. 2010) such as the phylogenetic marker 16S rRNA sequence, DNA–DNA hybridization, and DNA G+C content (Rosselló‐Mora 2006). These latter two tools have been considered a “gold standard” but are expensive and have poor reproducibility (Wayne et al. 1987). In recent years, the number of sequenced bacterial genomes has grown rapidly thanks to high‐throughput sequencing and MALDI‐TOF‐mass spectrometry (MALDI‐TOF‐MS) analysis. These have allowed the description of new bacterial genera and species. Recently, by adapting MALDI‐TOF to the routine identification of bacterial species, clinical isolated identification was made possible for the first time (La Scola and Raoult 2009; Seng et al. 2009, 2013). These methods provide a wealth of proteomic and genetic information (Tindall et al. 2010; Welker and Moore 2011).

Given that the current taxonomics rules do not integrate two of the recent revolutions in clinical microbiology, we proposed a new concept named taxonogenomics including these. As genome sequence provides access to the full genomic information of a strain at a reduced cost, we proposed in addition to the classic phenotypic description, to systematically add whole‐genome sequence and genome comparison with the closest species to describe a new isolate. Moreover, as MALDI‐TOF became the reference identification method in most clinical microbiology laboratories (Clark et al. 2013), we proposed systematically adding both MALDI‐TOF spectra and spectra comparison with the closest species to describe a new taxa. Using taxonogenomics, nine new bacteria have officially been considered as new genera and/or species in validation lists no. 153 and no. 155 (Kokcha et al. 2012; Lagier et al. 2012a,b,c,d, 2013a,b; Ramasamy et al. 2012; Roux et al. 2012; Hugon et al. 2013; Oren and Garrity 2014).

With both the description of the complete genomic sequencing and annotation, we present a summary classification and a set of features for *M. massiliensis* sp. nov. strain JC119^T^ (=CSUR P153 = DSM 26813) that was isolated from an African stool sample from Senegal (Table 1).

**Table 1:** Classification, general features, and project information of *Microvirga massiliensis* JC119^T^ according to the MIGS recommendations (Woese et al. 1990; Field et al. 2008)

MIGS ID	Property	Term	Evidence code^a^
	Current classification	Domain: *Bacteria*	TAS (Woese et al. 1990)
		Phylum: *Proteobacteria*	TAS (Garrity et al. 2005a)
		Class: *Alphaproteobacteria*	TAS (Oren and Garrity 2014; Garrity et al. 2005b)
		Order: *Rhizobiales*	TAS (Kuykendall 2005; Oren and Garrity 2014)
		Family: *Methylobacteriaceae*	TAS (Kanso and Patel 2003; Zhang et al. 2009; Weon et al. 2010; Ardley et al. 2012; Bailey et al. 2014; Radl et al. 2014; Reeve et al. 2014)
		Genus: *Microvirga*	TAS
		Species: *M. massiliensis*	IDA
		Type strain JC119^T^	IDA
	Gram stain	Negative	IDA
	Cell shape	Rod	IDA
	Motility	Nonmotile	IDA
Speculation	Nonsporulating	IDA	
	Temperature range	Mesophile	IDA
	Optimum temperature	37°C	IDA
MIGS‐6.3	Salinity	Unknown	
MIGS‐22	Oxygen requirement	Aerobic	IDA
	Carbon source	Unknown	IDA
	Energy source	Unknown	IDA
MIGS‐6	Habitat	Human gut	IDA
MIGS‐15	Biotic relationship	Free living	IDA
MIGS‐14	Pathogenicity	Unknown	NAS
	Biosafety level	2	
	Isolation	Human feces	
MIGS‐4	Geographic location	Dielmo, Senegal	IDA
MIGS‐5	Sample collection time	September 2010	IDA
MIGS‐4.1	Latitude	13.71667	IDA
MIGS‐4.1	Longitude	−16.41667	IDA
MIGS‐4.3	Depth	Surface	IDA
MIGS‐4.4	Altitude	34 m above sea level	IDA
MIGS‐31	Finishing quality	Noncontiguous finished	
MIGS‐28	Libraries used	Six paired‐end and two Shotgun 454; mate pair MiSeq	
MIGS‐29	Sequencing platform	454 GS FLX Titanium and Illumina	
MIGS‐31.2	Fold coverage	77×	
MIGS‐30	Assemblers	Newbler 2.8	
MIGS‐32	Gene calling method	Prodigal	
	GenBank Date of Release	November, 2014	
	NCBI project ID	PRJEB8433	
	EMBL accession	CDSD00000000	

a Evidence codes – IDA, inferred from direct assay; TAS, traceable author statement (i.e., a direct report exists in the literature); NAS, nontraceable author statement (i.e., not directly observed for the living, isolated sample, but based on a generally accepted property for the species, or anecdotal evidence). These evidence codes are from the Gene Ontology project (Ashburner et al. 2000).

## Materials and Methods

### Bacterial culture

#### Sample

In 2012, a stool sample was collected from a 16‐year‐old Senegalese man living in Dielmo, a rural area in Sine Saloum, Senegal. Signed informed consent was obtained from the patient. The study and the assent procedure were approved by the Ethics Committees of the Institut Fédératif de Recherche 48, Faculty of Medicine, Marseille, France, under agreement number 09‐022. After receipt, the stool sample was frozen at −80°C.

### Phenotypic characterization

#### Strain isolation

The stool sample was cultivated on MOD2 medium in aerobic conditions at 37°C for 48 h. The culture conditions included a modified 7h10 medium supplemented with sheep blood as previously described (Ghodbane et al. 2014).

#### Strain identification

All the colonies were identified using MALDI‐TOF MS, as described below. Colonies that exhibited a MALDI‐TOF‐MS score > 1.9 were considered as correctly identified, and colonies that exhibited a MALDI‐TOF‐MS score < 1.9 with spectra in the database were further characterized using 16S rRNA sequencing as previously described (Seng et al. 2010). If the similarity value between the 16S rRNA sequence was lower than 98.7%, we considered a new species without performing DNA–DNA hybridization as suggested by Stackebrandt and Ebers (2006).

#### Phenotypic properties

**Figure 1:**
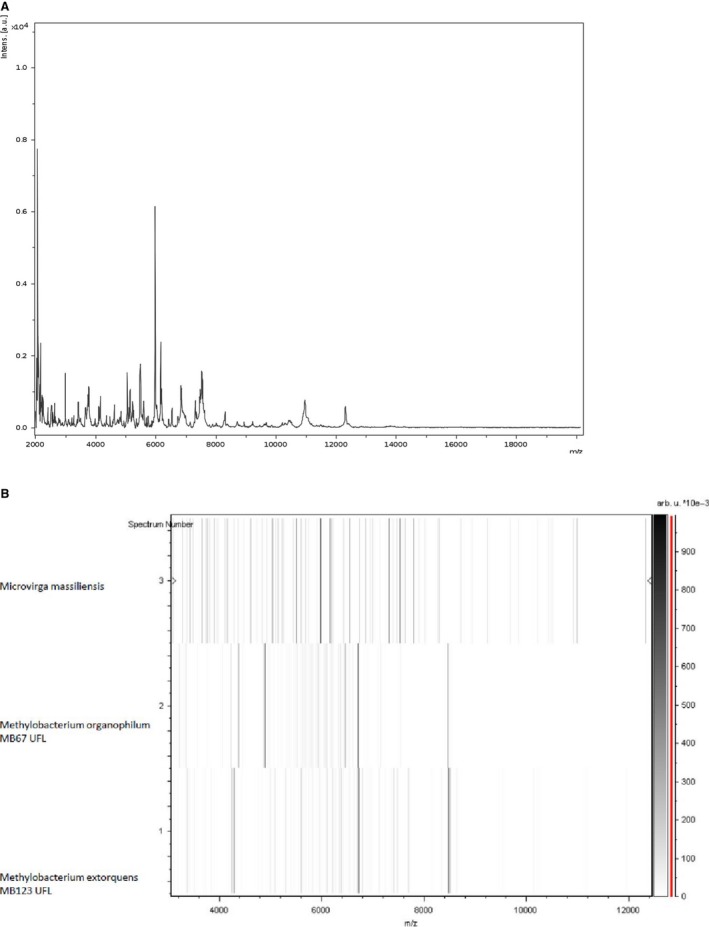
(A) Reference mass spectrum from *Microvirga massiliensis* JC119^T^. (B) Gel view comparing *M. massiliensis* strain to other *Methylobacteriaceae family*. The gel view displays the raw spectra of loaded spectrum files arranged in a pseudo‐gel‐like look. The *x*‐axis records the m/z value. The left *y*‐axis displays the running spectrum number originating from subsequent spectra loading. The peak intensity is expressed by a Gray scale scheme code. The color bar and the right *y*‐axis indicate the relation between the color a peak is displayed with and the peak intensity in arbitrary units. Displayed species are indicated on the left.

The main phenotypic characteristics such as Gram staining, motility, sporulation, catalase, and oxidase tests were performed as previously described (Schaeffer et al. 1965; Adler and Margaret 1967; Humble et al. 1977; Gregersen 1978). The chemical characteristics of the strain JC119^T^ were tested using API^®^ 20A, API^®^ ZYM, and API^®^ 50 CH strips (Biomerieux, Marcy‐l'Etoile, France). Growth temperatures were tested at 25°C, 30°C, 37°C, 45°C, and 55°C, respectively. Strain growth was tested under aerobic conditions, with or without 5% CO_2,_ and under anaerobic and microaerophilic conditions using GENbag anaer and GENbag microaer systems, respectively (Biomerieux). For electron microscopy, we observed colonies using a Morgani 268D (FEI, Limeil‐Brevannes, France) at an operating voltage of 60 kV. Antibiotic disks of vancomycin (30 μg), rifampicin (30 μg), doxycycline (30 μg), erythromycin (15 μg), amoxicillin (25 μg), nitrofurantoin (300 μg), gentamicin (15 and 500 μg), ciprofloxacin (5 μg), amoxicillin (20 μg) with clavulanic acid (10 μg), penicillin G (10 μg), trimethoprim (1.25 μg) with sulfamethoxazole (23.75 μg), oxacillin (5 μg), imipenem (10 μg), tobramycin (10 μg), metronidazole (4 μg), and amikacin (30 μg) were purchased from Biomerieux. In vitro susceptibility testing of these antibiotics was performed by the disk diffusion method following EUCAST recommendations (Matuschek et al. 2014). Results were interpreted using Eucast 2015 clinical breakpoints (http://www.eucast.org/clinical_breakpoints/). MALDI‐TOF‐MS protein analysis was carried out using a Microflex spectrometer (Bruker Daltonics, Leipzig, Germany) as previously described (Seng et al. 2009). Briefly, one isolated bacterial colony was transferred with a pipette tip from a culture agar plate and spread as a thin film on a MSP 96 MALDI‐TOF target plate (Bruker). After isolation, 12 distinct deposits from 12 isolated colonies were tested for strain JC119^T^. We superimposed each smear with 2 μL of matrix solution (saturated solution of α‐cyano‐4‐hydroxycinnamic acid) in 2.5% trifluoroacetic acid, 50% acetonitrile and allowed to dry for 5 min. We recorded the spectra in the positive linear mode for the mass range of 2000–20,000 Da (parameter settings: ion source 1 (ISI), 20 kV; IS2, 18.5 kV; lens, 7 kV). Through variable laser power, a spectrum was obtained after 240 shots. Per spot, the acquisition time was between 30 and 60 s. The strain JC119^T^ spectra were imported into the MALDI BioTyper software (version 3.0, Bruker) and analyzed by standard pattern matching (with default parameter settings) against the main spectra of 2843 bacteria. The identification method included m/z from 3000 to 15,000 Da. For every spectrum, we compared spectra in the database with a maximum of 100 peaks. From the resulting score, we could identify tested species, where a score ≥ 1.9 enabled identification at the species level with a validly published species. For strain JC119^T^, no significant MALDI‐TOF score was obtained against the database, suggesting that this isolate was not a member of a known species. We added the spectrum from strain JC119^T^ to our database (Fig. 1A). Finally, the gel view was used to demonstrate the spectral differences with other members of the *Methylobacteriaceae* family (Fig. 1B).

### Genome sequencing and assembly

Genomic DNA of *M. massiliensis* was sequenced on a 454 sequencer with two methods: paired‐end and shotgun and on a MiSeq sequencer (Illumina, Inc., San Diego, CA) with mate pair strategy; 450 μL of bacterial suspension was diluted in 2 mL TE buffer for lysis treatment: a lysozyme incubation of 30 min at 37°C followed by an overnight proteinase K incubation at 37°C. The DNA was purified by three phenol–chloroform extractions and ethanolic precipitation at −20°C overnight. After centrifugation, the DNA was resuspended in 180 μL TE buffer. The concentration was measured by the Quant‐it Picogreen kit (Invitrogen) on the Genios_Tecan fluorometer at 55.70 ng/μL. This project was sequenced through two NGS technologies 454‐Roche GSFLX Titanium GS and Illumina MiSeq. A shotgun and 3‐kb paired‐end libraries were pyrosequenced on the 454_Roche_Titanium. This project was loaded twice on a 1/4 region for the shotgun library and for the paired‐end library: once on a full PTP PicoTiterPlate and four times on a 1/4 region on PTP PicoTiterPlates. The libraries were constructed according to the 454_Titanium and manufacturer protocols. The shotgun library was constructed with 500 ng of DNA as described by the manufacturer Roche with the GS Rapid library Prep kit with a final concentration at 1.35e^09^ by the Quant‐it Ribogreen kit (Invitrogen) on the Genios_Tecan fluorometer. The paired‐end library was constructed from 5 μg of DNA. The DNA was mechanically fragmented on the Hydroshear device (Digilab, Holliston, MA) with an enrichment size at 3–4 kb. The DNA fragments were visualized through the Agilent 2100 BioAnalyzer (Agilent Technologies Inc., Santa Clara, CA) on a DNA labchip 7500 with an optimal size of 2.950 kb. Circularization and nebulization were performed and generated a pattern with an optimal at 371 bp. After PCR amplification through 14 cycles followed by double size selection, the single‐stranded paired‐end libraries were then quantified on the Quant‐it Ribogreen kit (Invitrogen) on the Genios_Tecan fluorometer at 140 pg/μL. The library concentration equivalence was calculated at 6.92e^08^. The two libraries were stocked at −20°C until use. The shotgun library was clonal‐amplified with 3 cpb in 3 SV emPCR and the 3‐kb paired‐end library was amplified with lower cpb (0.5 cpb) in 4 SV emPCR and by a bigger preparation of enriched beads at 0.25, 0.5, and 0.75 cpb with the GS Titanium MV emPCR Kit (Lib‐L) v2. The yield of the emPCR was 16.83% for the shotgun and for two clonal amplification of the 3‐kb paired‐end library 9.65% in SV reactions and 6.79%, 14.31%, and 14.56%, respectively, in MV reactions. These yields were measured according to the quality expected by the range of 5% to 20% from the Roche procedure. The two libraries were loaded on the GS Titanium PicoTiterPlates PTP Kit 70 × 75 sequenced with the GS Titanium Sequencing Kit XLR70. The runs were performed overnight and then analyzed on the cluster through the gsRunBrowser and gsAssembler_Roche; 904 Mb was generated through passed filters reads with a length average of 299 bp. A second DNA extraction was performed. Three petri dishes were spread and resuspended in 6 × 100 μL of G2 buffer. First, mechanical lysis was performed by glass powder on the Fastprep‐24 device (sample preparation system) from MP Biomedicals for 2 × 20 s. DNA was then incubated for a lysozyme treatment (30 min at 37°C) and extracted through the BioRobot EZ 1 Advanced XL (Qiagen, Hilden, Germany)). The gDNA was quantified by a Qubit assay with the high sensitivity kit (Life Technologies, Carlsbad, CA) to 118 ng/μL.

Genomic DNA of *M. massiliensis* was sequenced on the MiSeq Technology (Illumina) with the mate pair strategy. The gDNA was barcoded in order to be mixed with 11 other projects with the Nextera mate pair sample prep kit (Illumina). The mate pair library was prepared with 1 μg of genomic DNA using the Nextera mate pair Illumina guide. The genomic DNA sample was simultaneously fragmented and tagged with a mate pair junction adapter. The pattern of the fragmentation was validated on an Agilent 2100 BioAnalyzer (Agilent Technologies Inc.) with a DNA 7500 labchip. The DNA fragments ranged in size from 1 kb up to 10 kb with an optimal size at 4.81 kb. No size selection was performed and 670 ng of tagmented fragments was circularized. The circularized DNA was mechanically sheared to small fragments with an optimal at 667 bp on the Covaris device S2 in microtubes (Covaris, Woburn, MA). The library profile was visualized on a High Sensitivity Bioanalyzer LabChip (Agilent Technologies Inc.). The libraries were normalized at 2 nM and pooled. After a denaturation step and dilution at 12 pM, the pool of libraries was loaded onto the reagent cartridge and then onto the instrument along with the flow cell. Automated cluster generation and sequencing run were performed in a single 39 h run in a 2 × 251 bp. Total information of 8.3 Gb was obtained from a 947 K/mm^2^ cluster density with a cluster passing quality control filters of 99% (18,112,000 clusters). Within this run, the index representation for *Microvirga* was determined to be 8.57%. The 1,398,916 paired reads were filtered according to the read qualities.

The total of 2,915,781 reads produced by six 454 paired‐end run and two 454 shotgun of *M. massiliensis* JC119^T^ were assembled with Newbler version 2.8 which generated a genome size of 9.3 Mb with an average of 77× coverage of the genome. For increased quality of the genome, we mapped reads from MiSeq sequencer against this genome using CLC workbench software (CLC bio, Aarhus, Denmark).

### Genome annotation and comparison

Open reading frames (ORFs) were predicted using Prodigal (Hyatt et al. 2010) with default parameters. However, the predicted ORFs were excluded if they were spanning a sequencing gap region (containing N). The predicted bacterial protein sequences were searched against Clusters of Orthologous Groups (COG) databases (Tatusov et al. 2001) using BLASTP (Altschul et al. 1990) (*E*‐value 1e^−03^, coverage 70%, and identity percent 30%). If no hit was found, the search was performed against the NR database using BLASTP with the same parameters as before. If the sequence lengths were smaller than 80 amino acids, we used an *E*‐value of 1e^−05^. The tRNA genes were found by the tRNAScanSE tool (Lowe and Eddy 1997), whereas ribosomal RNAs (rRNAs) were found by using RNAmmer (Lagesen et al. 2007). Lipoprotein signal peptides and the number of transmembrane helices were predicted using Phobius (Käll et al. 2004). ORFans were identified if all the BLASTP performed did not give positive results (*E*‐value smaller than 1e^−03^ for ORFs with a sequence size higher than 80 aa or *E*‐value smaller than 1e^−05^ for ORFs with sequence lengths smaller than 80 aa). Similar parameter thresholds have already been used in previous work to define ORFans (Yin and Fischer 2006, 2008).

Based on 16S rDNA of *M. massiliensis* (JF824802) and closely related species, sequences were aligned using CLUSTALW, and phylogenetic tree was obtained using the maximum‐likelihood method within the MEGA 6.06 software (Tamura et al. 2013). Numbers at the nodes are bootstrap values obtained by repeating the analysis 500 times to generate a majority consensus tree. We performed a BLASTP (Altschul et al. 1990) against the database of microbial polyketide and nonribosomal peptide gene clusters (Conway and Boddy 2013) for the predicted number of polyketide synthase (PKS)‐ and nonribosomal peptide synthetase (NRPS)‐encoding genes, and we keep only the best hit for each protein.

For the genomic comparison, we used *M. massiliensis* strain JC119^T^ (CDSD00000000), *Microvirga aerilata* strain BSC39 (JPUG00000000), *Microvirga flocculans* strain ATCC BAA‐817 (JAEA00000000), *M. lotononidis* strain WSM3557 (AJUA00000000), and *Microvirga lupini* strain Lut6 (AZYE00000000) genomes. Orthologous proteins were identified using the Proteinortho software, version 1.4 (Lechner et al. 2011), using a 30% protein identity and 1e^−05^ *E*‐value. The average genomic identity of orthologous gene sequences (AGIOS) between compared genomes was determined using the Needleman–Wunsch algorithm global alignment technique. Artemis (Carver et al. 2005) was used for data management, and DNA Plotter (Carver et al. 2009) was used for the visualization of genomic features. The mauve alignment tool was used for multiple genomic sequence alignment and visualization (Darling et al. 2010). In silico DNA–DNA hybridization (DDH) (Richter and Rosselló‐Móra 2009) was performed with the genomes previously cited. *M. Massiliensis* genome was locally aligned 2‐by‐2 using BLAT algorithm (Kent 2002; Auch et al. 2010) against each of the selected genomes, and DDH values were estimated from a generalized linear model (Meier‐Kolthoff et al. 2013).

### Pulsed field gel electrophoresis (PFGE)

Plug preparation and treatment and pulsed field gel electrophoresis (PFGE) of bacterial DNA were performed as previously described by Raoult et al. (2004). *Spe*I restriction enzymes (Life Technologies) were used to migrate genomic DNA.

Each agarose block and molecular weight markers (Low Range PFG Marker; Biolabs, New England, New England Biolabs, Ipswich, Massachusetts, USA) were placed in the well of a 1% PFGE agarose gel (Sigma, St. Louis, Missouri, USA) in 0.5× TBE.

The pulsed field gel separation (Fig. S1) was made on a CHEF–DR II apparatus (Bio‐Rad Laboratories Inc., Hercules, California, USA) with pulses ranging from 5 to 50 s at a voltage of 5 V/cm and switch angle of 120° for 20 h at 14°C. Gels were either stained with ethidium bromide and analyzed using a Gel‐Doc 2000 system (Bio‐Rad Laboratories Inc.) or used to prepare Southern blots.

### DNA labeling and hybridization

Resolved uncut and digested genomic DNA processed by PFGE were treated and transferred onto Hybond N+ (GE Healthcare, Little Chalfont, UK) with the vacuum blotter (model 785, Bio‐Rad Laboratories Inc.) and UV‐cross‐linked for 2 min. The blots were then hybridized against the DIG‐labeled probes (scaffolds 24 and 35) as recommended by the manufacturer DIG‐System (Roche Diagnostics, Meylan, France) except that the hybridized probe was detected using a horseradish peroxidase‐conjugated monoclonal mouse anti‐digoxin (Jackson Immunoresearch, West Grove, Pennsylvania, USA, 1:20,000). After washings, blots were revealed by chemiluminescence assays (ECL; GE Healthcare). The resulting signal was detected on Hyperfilm^™^ ECL (GE Healthcare) by using an automated film processor Hyperprocessor^™^ (GE Healthcare).

## Results

### Phenotypic characteristics

**Figure 2:**
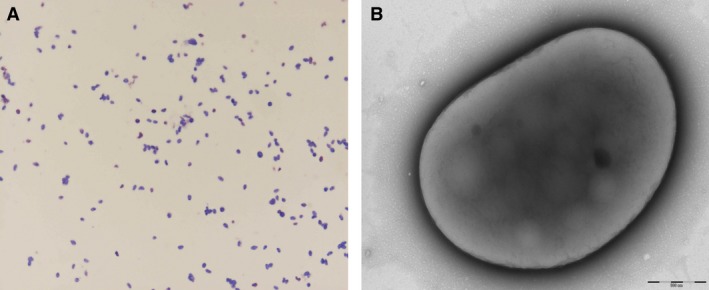
(A) Gram straining of *Microvirga massiliensis* JC119^T^. (B) Transmission electron microscopy of *M. massiliensis* JC119^T^, taken using a Morgagni 268D (Philips Amsterdam, Netherlands) at an operating voltage of 60 kV. The scale represents 500 nm.

Gram staining showed gram‐negative bacilli (Fig. 2A). The motility test was negative. Cells grown on agar were nonsporulated and had a mean diameter of 2.28 μm (Fig. 2B). Catalase was positive, and oxidase was negative.

Using API^®^ 20NE (BioMerieux), positive reactions were observed to reduce nitrite to nitrate and glucose fermentation. Using API^®^ ZYM strips (BioMerieux), positive reactions were observed for esterase (C4), esterase lipase (C8), leucine arylamidase, cysteine arylamidase, trypsin, acid phosphatase, and naphthol‐AS‐BI‐phosphohydrolase. Using an API^®^ 50 CH (BioMerieux), positive reactions were recorded for d‐trehalose hydrolysis after 48 h. These results are summarized in Tables 2, 3, 4.

**Table 2:** *Microvirga massiliensis* sp. nov. reactions results with API^®^ 20NE

Active components	Result
Potassium nitrate	+
l‐Tryptophan	−
d‐Glucose	+
l‐Arginine	−
Urea	−
Esculine	−
Gelatin	−
4‐Nitrophenyl α‐d‐galactopyranoside	−
d‐Glucose	−
l‐Arabinose	−
d‐Mannose	−
d‐Mannitol	−
N‐Acetyl‐glucosamine	−
d‐Maltose	−
Potassium gluconate	−
Capric acid	−
Adipic acid	−
Malic acid	−
Trisodium citrate	−
Phenylacetic acid	−
Oxidase	−

**Table 3:** *Microvirga massiliensis* sp. nov. reactions results with API^®^ ZYM

Enzyme assayed for	Result
Alkaline phosphatase	−
Esterase (C4)	+
Esterase lipase (C8)	+
Lipase (C14)	−
Leucine arylamidase	+
Valine arylamidase	−
Cysteine arylamidase	+
Trypsin	+
α‐Chymotrypsin	−
Acid phosphatase	+
Naphthol‐AS‐BI‐phosphohydrolase	+
α‐Galactosidase	−
β‐Galactosidase	−
β‐Glucuronidase	−
α‐Glucosidase	−
β‐Glucosidase	−
N‐Acetyl‐β‐glucosaminidase	−
α‐Mannosidase	−
α‐Fucosidase	−

**Table 4:** *Microvirga massiliensis* sp. nov. reactions results with API^®^ 50CH

Active components	Result
Glycerol	−
Erythritol	−
d‐Arabinose	−
l‐Arabinose	−
d‐Ribose	−
d‐Xylose	−
l‐Xylose	−
d‐Adonitol	−
Methyl β‐d‐xylopyranoside	−
d‐Galactose	−
d‐Glucose	−
F‐Fructose	−
d‐Mannose	−
l‐Sorbose	−
l‐Rhamnose	−
Dulcitol	−
Inositol	−
d‐Mannitol	−
d‐Sorbitol	−
Methyl α‐d‐mannopyranoside	−
Methyl α‐d‐glucopyranoside	−
N‐Acetylglucosamine	−
Amygdaline	−
Arbutine	−
Esculine	−
Salicine	−
d‐Cellobiose	−
d‐Maltose	−
d‐Lactose	−
d‐Melibiose	−
d‐Saccharose	−
d‐Trehalose	+
Inuline	−
d‐Melezitose	−
d‐Raffinose	−
Amidon	−
Glycogene	−
Xylitol	−
Gentiobiose	−
d‐Turanose	−
d‐Lyxose	−
d‐Tagatose	−
d‐Fucose	−
d‐Arabitol	−
l‐Arabitol	−
Potassium gluconate	−
Potassium 2‐ketogluconate	−
Potassium 5‐ketogluconate	−

*Microvirga massiliensis* strain JC119^T^ was susceptible for rifampicin, doxycycline, erythromycin, amoxicillin, gentamicin, ciprofloxacin, ceftriaxone, amoxicillin and clavulanic acid, penicillin G, imipenem, tobramycin, metronidazole, amikacin and resistant to vancomycin and nitrofurantoin.

When compared to the species *M. aerophila, M. aerilata*,* Methylobacterium nodulans*, and *Methylobacterium populi*,* M. massiliensis* exhibits phenotypic characteristics as detailed in Table 5.

**Table 5:** Differential characteristics of *Microvirga massiliensis* sp. nov., strain JC119^T^, *Microvirga aerophila* strain 5420S‐12^T^ (Weon et al. 2010), *Microvirga aerilata* strain 5420S‐16^T^ (Weon et al. 2010), *Methylobacterium nodulans* strain ORS 2060^T^ (Jourand et al. 2004), and *Methylobacterium populi* strain BJ001^T^ (Aken et al. 2004)

Properties	*M. massiliensis*	*M. aerophila*	*M. aerilata*	*M. nodulans*
Cell diameter (μm)	2.28	0.8–1.1	1.2–1.5	0.5–1
Oxygen requirement	Aerobic	Aerobic	Aerobic	Aerobic
Gram stain	Negative	Negative	Negative	Negative
Motility	−	−	−	±
Endospore formation	−	−	−	−
*Production of*				
Alkaline phosphatase	−	−	+	NA
Acid phosphatase	+	+	+	NA
Catalase	+	NA	NA	NA
Oxidase	−	NA	NA	NA
Nitrate reductase	+	NA	NA	+
Urease	−	NA	NA	+
α‐Galactosidase	−	−	−	NA
β‐Galactosidase	−	−	−	−
β‐Glucuronidase	−	−	−	NA
α‐Glucosidase	−	−	−	NA
β‐Glucosidase	−	−	−	−
Esterase	+	+	+	NA
Esterase lipase	+	−	+	NA
Naphthol‐AS‐BI‐phosphohydrolase	+	+	+	NA
N‐Acetyl‐β‐glucosaminidase	−	−	−	NA
α‐Mannosidase	−	−	−	NA
α‐Fucosidase	−	−	−	NA
Leucine arylamidase	+	−	+	NA
Valine arylamidase	−	−	−	NA
Cystine arylamidase	+	−	−	NA
α‐Chymotrypsin	−	−	−	NA
Trypsin	+	−	+	NA
*Acid from*				
l‐Arabinose	−	NA	NA	+
d‐Mannose	−	NA	NA	+
d‐Mannitol	−	NA	NA	−
d‐Trehalose	+	NA	NA	+
d‐Mannose	−	NA	NA	+
Habitat	Human gut	Air	Air	*C. podocarpa*

NA, data not available.

### Genomic characteristics

**Figure 3:**
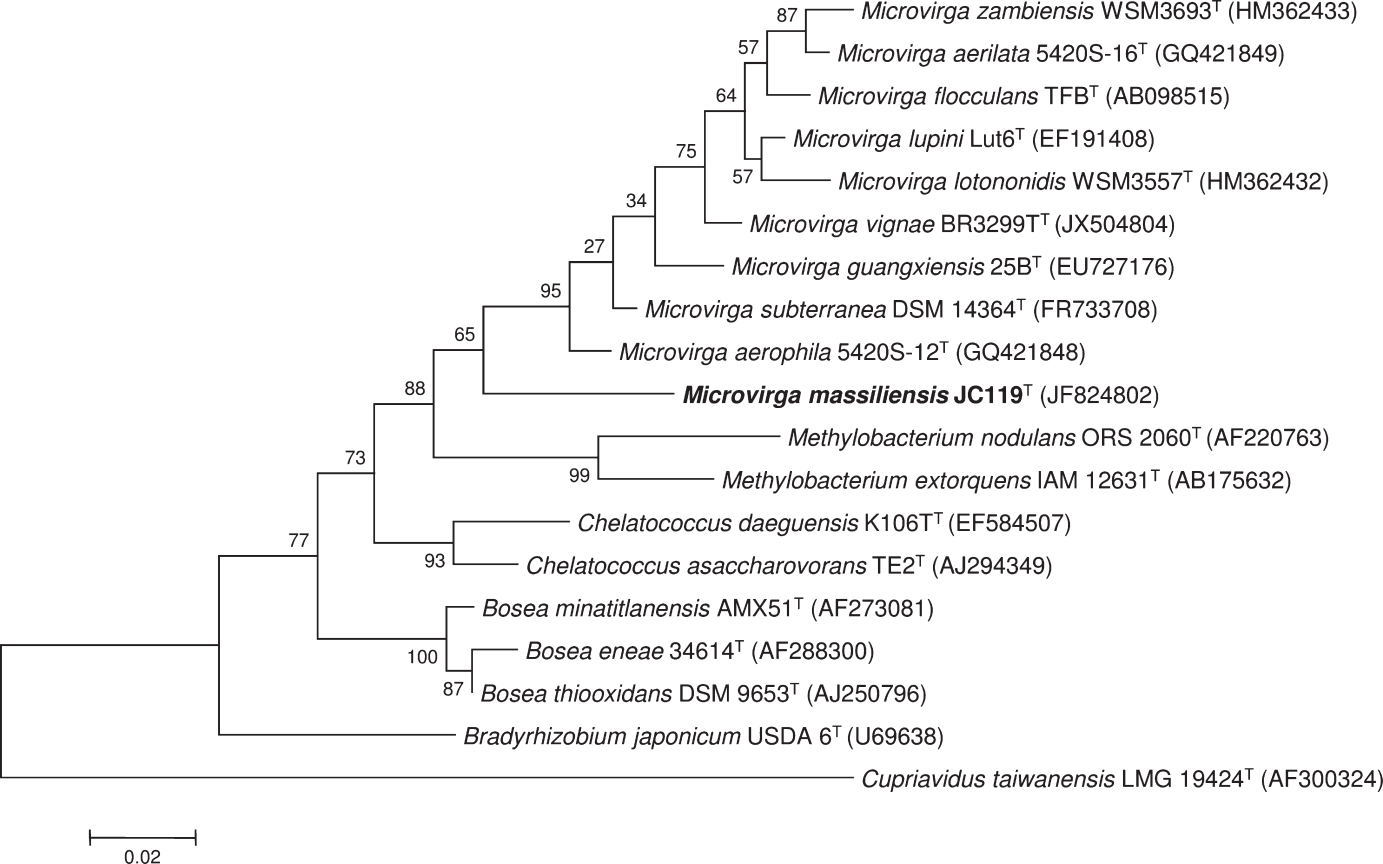
Phylogenetic tree highlighting the position of *Microvirga massiliensis* JC119^T^ relative to other type strains within the *Microvirga* genus and closely related species. The strains and their corresponding accession number for 16S rRNA genes are indicated in parentheses. *Cupriavidus taiwanensis* strain LMG 19424^T^ was used as outgroup. The scale bar represents a 2% nucleotide sequence divergence.

**Figure 4:**
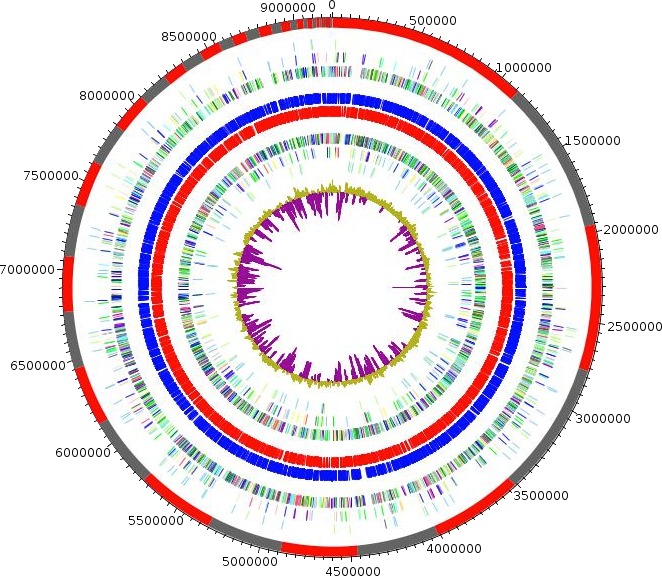
Circular representation of the *Microvirga massiliensis* strain JC119^T^ genome. From outside to the center: contigs (red/gray), COG category of genes on the forward strand (three circles), genes on forward strand (blue circle), genes on the reverse strand (red circle), COG category on the reverse strand (three circles), GC content.

Based on 16S rDNA, the *M. massiliensis* strain JC119^T^ (accessory number JF824802) exhibited, among other, 95% sequence identity with *M. lotononidis* WSM3557^T^ (accessory number HM362432) (Ardley et al. 2012). We summarized the closest strains of *M. massiliensis* based on their sequence identity percentage in Table S1. The phylogenetic tree highlights the position of *M. massiliensis* JC119^T^ (Fig. 3) in relation to other type strains within the *Microvirga* genus and closely related species. The genome of *M. massiliensis* JC119^T^ is 9,207,211 bp long with 63.28% GC content (Fig. 4). It is composed of 50 scaffolds (accession number LN811350–LN811399) (composed of 365 contigs) with one plasmid. Table 1 shows the project information and its association with MIGS version 2.0 compliance (Field et al. 2008). Of the 8762 predicted genes, 8685 were protein‐coding genes, 56 tRNA genes, and 21 rRNA genes. A total of 5323 genes (61.29%) were assigned as putative function (by COGs or by NR blast); 1500 genes were identified as ORFans (17.27%). The remaining genes were annotated as hypothetical proteins. We have predicted the number of rRNA by mapping. We mapped all reads against the rRNA operon and all reads against the 50 scaffolds. The average coverage of the rRNA operon is seven times higher than the average coverage of the genome so this genome has seven genes that are 16S rRNA, seven genes that are 23S rRNA, and seven genes that are 5S rRNA. The circular plasmid sequence is complete with 73,638 bp long and was found by PFGE.

We have predicted 44 PKS/NRPS summarized Table 6. The distribution of genes into COGs functional categories is presented in Table 7. We found that the 263 genes belonging to the L category especially represented genes encoding for transposases, which are larger than *M. flocculans* (140 genes) but smaller than those of *M. aerilata, M. lotononidis,* and *M. lupini* (288, 419, and 583 genes, respectively). The increase in transposable elements is related to the size of bacterial genome (Touchon and Rocha 2007; Chénais et al. 2012; Iranzo et al. 2014). Additionally, two scaffolds (scaffolds 43 and 48) were expected to contain a large number of transposable elements (Pray 2008) because their coverage represented almost 350× and 250×, respectively, in comparison with the average coverage of the entire genome, that is, 50×, that allows us to say that the number of transposases is underestimated. We can estimate that the most real number of transposable elements would be five to seven times more. Moreover, we found twelve genes to be larger than 5000 nucleotides, one of which was larger than 14 kb and corresponded to a predicted function of a rearrangement hotspot (RHS) repeat protein. RHS proteins contain extended repeats, which are involved in recombination (Koskiniemi et al. 2013).

**Table 6:** NRPS/PKS predicted for *Microvirga massiliensis*

PKS/NRPS on proteome (best hit)	
Streptomyces avermitilis MA‐4680 (NC_003155)	a
Streptomyces sp. FR‐008 (AY310323)	
Mycobacterium avium 104 (CP000479)	
Streptomyces griseoviridis (AB469822)	a
Sorangium cellulosum (AF210843)	
Bacillus amyloliquefaciens FZB42 (AJ576102)	
Micromonospora chalcea (EU443633)	a
Streptomyces coelicolor A3(2) (NC_003888)	
Streptomyces venezuelae ATCC 10712 (FR845719)	a
Saccharopolyspora erythraea NRRL 2338 (NC_009142)	
Acidobacteria bacterium A11 (JF342591)	a
Sorangium cellulosum (DQ897667)	
Myxococcus xanthus DK 1622 (CP000113)	a
Mycobacterium avium subsp. paratuberculosis K‐10 (AE016958)	
Nocardia farcinica IFM 10152 (NC_006361)	a
Mycobacterium gilvum PYR‐GCK (NC_009338)	
Chitinophaga sancti (HQ680975)	
Sorangium cellulosum (HE616533)	a
Mycobacterium tuberculosis CDC 1551 (NC_002755)	
Mycobacterium sp. MCS (CP000384)	
Sorangium cellulosum (AM407731)	
Streptomyces sp. MP39‐85 (FJ872525)	
Streptomyces antibioticus (FJ545274)	
Tistrella mobilis KA081020‐065 (NC_017956)	
Streptomyces ambofaciens ATCC 23877 (AM238664)	
Mycobacterium smegmatis str. MC2 155 (CP000480)	
Thermomonospora curvata DSM 43183 (NC_013510)	
Microcystis aeruginosa PCC 7806 (AF183408)	

a This best hit corresponded to several genes (to two from seven) in *M. massiliensis*.

**Table 7:** Number of genes associated with the 25 general COG functional categories

Code	Value	% value	Description
J	195	2.25	Translation
A	0	0	RNA processing and modification
K	359	4.13	Transcription
L	263	3.03	Replication, recombination, and repair
B	9	0.1	Chromatin structure and dynamics
D	29	0.33	Cell cycle control, mitosis, and meiosis
Y	0	0	Nuclear structure
V	66	0.76	Defense mechanisms
T	283	3.26	Signal transduction mechanisms
M	263	3.03	Cell wall/membrane biogenesis
N	67	0.77	Cell motility
Z	0	0	Cytoskeleton
W	0	0	Extracellular structures
U	80	0.92	Intracellular trafficking and secretion
O	184	2.12	Post‐translational modification, protein turnover, chaperones
C	347	4	Energy production and conversion
G	423	4.87	Carbohydrate transport and metabolism
E	788	9.07	Amino acid transport and metabolism
F	88	1.01	Nucleotide transport and metabolism
H	175	2.01	Coenzyme transport and metabolism
I	256	2.95	Lipid transport and metabolism
P	368	4.24	Inorganic ion transport and metabolism
Q	229	2.64	Secondary metabolites biosynthesis, transport, and catabolism
R	802	9.23	General function prediction only
S	525	6.04	Function unknown
–	3724	42.88	Not in COGs

**Figure 5:**
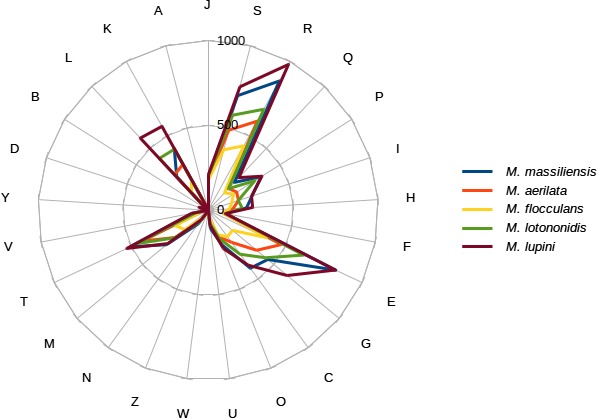
Distribution of functional classes of predicted genes in the genomes from *Microvirga massiliensis, Microvirga aerilata, Microvirga flocculans, Microvirga lotononidis, and Microvirga lupini* chromosomes*,* according to the COG category.

We compared the genome of *M. massiliensis* strain JC119^T^ to *M. aerilata* strain BSC39, *M. flocculans* strain ATCC BAA‐817, *M. lotononidis* strain WSM3557^T^, and *M. lupini* strain Lut6^T^ (Table 8). The draft genome sequence of *M. lupin*i is larger than those of *M. massiliensis*,* M. lotononidis*,* M. aerilata*, and *M. flocculans* (9.7, 9.3, 7.1, 5.7, and 4.1 MB, respectively). The G+C content of *Microvirga* species ranged from 61.6 to 64.6 (Table 8). The gene content of *M. massiliensis* (8685) is smaller than that of *M. lupini* (9865), but larger than those of *M. lotononidis, M. aerilata,* and *M. flocculans* (6991, 5571, and 3835, respectively). The distribution of genes into COG categories was similar, but not identical in all compared genomes (Fig 5). In addition, *M. massiliensis* shared 2575, 2275, 2713, and 2729 orthologous genes with *M. aerilata, M. flocculans, M. lotononidis,* and *M. lupini*, respectively (Table 9). The AGIOS values ranged from 80.19 to 82.97 among compared *Microvirga* species except *M. massiliensis*. When compared to other species, the AGIOS value ranged from 67.91% with *M. lupini* to 69.45% with *M. flocculans*. The DDH was of 21.7% ±2.33 with *M. lupini*, 21% ±2.33 with *M. aerilata*, 20.90% ±2.33 with *M. flocculans,* and 20.30% ±2.32 with *M. lotononidis*. These data confirm *M. massiliensis* as a unique species.

**Table 8:** Comparative genomic features of *Microvirga massiliensis, Microvirga aerilata, Microvirga flocculans, Microvirga lotononidis,* and *Microvirga lupini*

	*M. massiliensis*	*M. aerilata*	*M. flocculans*	*M. lotononidis*	*M. lupini*
Genome size (Mb)	9.3	5.7	4.1	7.1	9.7
DNA G+C content (%)	63.3	63.1	64.6	62.9	61.6
Protein‐coding genes	8685	5571	3835	6991	9865
rRNA	21	13	5	6	3
tRNA	56	58	48	55	57

**Table 9:** Genomic comparison of *Microvirga massiliensis* and four other *Microvirga* species

	*M. aerilata*	*M. flocculans*	*M. lotononidis*	*M. lupini*	*M. massiliensis*
*M. aerilata*	**5571**	2665	3131	3163	2575
*M. flocculans*	82.21	**3835**	2761	2691	2275
*M. lotononidis*	80.83	81.67	**6992**	3305	2713
*M. lupini*	80.19	80.8	82.97	**9865**	2729
*M. massiliensis*	68.69	69.45	68.74	67.91	**8685**

Numbers of orthologous proteins shared between genomes (upper right) and AGIOS values (lower left); bold numbers indicate the numbers of proteins per genome.

## Discussion

The *Methylobacteriaceae* family is comprised of four genera: *Meganema*,* Methylobacterium*,* Psychroglaciecola,* and *Microvirga*. Currently, the genus *Microvirga* contains nine species (Kanso and Patel 2003; Zhang et al. 2009; Weon et al. 2010; Ardley et al. 2012; Radl et al. 2014). The first species of the *Microvirga* genus was *M. subterranea* strain Fail4^T^ (Kanso and Patel 2003) and was first deposited as *Corbulabacter subterraneus*. Species of the genus *Microvirga* are mostly found in soil. To the best of our knowledge, no *Microvirga* strain has previously been isolated from humans. *M. massiliensis* strain JC119^T^ is the type strain of *M. massiliensis* sp. nov., a new species of the genus *Microvirga*. The genome of *M. massiliensis* JC119^T^ is 9,207,211 bp long, representing the largest genome of a bacteria isolated from a human sample (Lagier et al. 2012a,b,c,d). It ranks 139th among the largest bacterial genomes according to the Genomes OnLine Database (GOLD) (Kyrpides 1999). All these bacteria whose genome is larger than *M. massiliensis* are found in the environment (soil, ocean, sand, and landfill). Different characteristics of this bacterium can explain a broad genome. The proportion of no detectable homologs (ORFans) from the gene prediction is about 17%, which corresponds to 1500 ORFans. The number of noncoding genes is important. This genome has 77 RNA: 56 tRNA and 21 rRNA. The number of RNA appears to be related to genome size (Klappenbach et al. 2000). For bacterial genomes, the copy number of rRNA operons varies from 1 to 15, for example, for *Clostridium paradoxum* (Rainey et al. 1996). There are also a large number of transposases that create repeat elements in this genome. The abundance of transposable elements correlates positively with genome size (Touchon and Rocha 2007; Chénais et al. 2012; Iranzo et al. 2014).

Culture remains a critical step of microbiology (Lagier et al. 2015). However, thanks to the multiplication of culture conditions, culturomics increased the knowledge of the human gut microbiota repertoire by 77% and thus helps to decipher the “dark matter” (Lagier et al. 2012a,b,c,d, 2015). Culturomics allows the generation of stable and robust data such as pure culture and genome sequencing by contrast with metagenomics, which shows a significant lack of reproducibility among laboratories (Angelakis et al. 2012).

Taxonogenomics allows the consideration of proteome and genome sequences, which must be a part of the taxonomic description of bacteria. To describe a new bacterial species, taxonogenomics uses both phenotypic and genotypic data (Ramasamy et al. 2012; Lagier et al. 2015). On the basis of taxonogenomics, we formally propose the creation of *M. massiliensis* sp. nov. containing the strain JC119^T^.

### Description of *Microvirga massiliensis* sp nov

*Microvirga massiliensis* (mas.si.li.en'sis. L. masc. adj. massiliensis of Massilia, the Latin name of Marseille, France, where *M. massiliensis* was isolated) is a gram‐negative, oxidase‐negative, and catalase‐positive bacterium. This bacterium is an aerobic, nonmotile rod, and non‐spore‐forming, individual cell exhibiting a diameter of 2.28 μm. This bacterium is positive for nitrate reductase, d‐glucose, esterase, esterase lipase, leucine arylamidase, cysteine arylamidase, trypsin, acid phosphatase, and naphthol‐AS‐BI‐phosphohydrolase. This bacterium is susceptible to rifampicin, doxycycline, erythromycin, amoxicillin, gentamicin, ciprofloxacin, ceftriaxone, amoxicillin with clavulanic acid, penicillin, imipenem, tobramycin, metronidazole, and amikacin. The range of temperature growth is 28–55°C (with an optimum at 37°C). The potential pathogenicity of the type strain JC119^T^ (=CSUR P153 = DSM 26813) is unknown but was isolated from the stool specimen from a Senegalese male, in Marseille (France), using culturomics (Lagier et al. 2012a,b,c,d; Dubourg et al. 2014). The G+C content of the genome is 63.28%. The partial 16S rRNA sequence of *M. massiliensis* was deposited in GenBank under the accession number JF824802. The whole‐genome sequence of *Microvirga massiliensis* strain JC119^T^ (=CSUR P153 = DSM 26813) has been deposited in EMBL under accession numbers CDSD01000001–CDSD01000365 for contigs and LN811350–LN811399 for scaffolds.

## Funding Information

No funding information provided.

## Conflict of Interest

None declared.

## Supporting information


**Figure S1.** Pulsed field gel electrophoresis (PFGE) and Southern blot (A) PFGE of intact genomic DNA (ND) and SpeI‐digested DNA from *Microvirga massiliensis*. Electrophoresis was performed in 1% agarose in 0.5× TBE buffer, and the pulse time was ramped from 5 to 50 s for 20 h at a voltage of 5 V/cm for 20 h at 14°C. Gel was stained with ethidium bromide. Low Range PFG Marker (Biolabs, New England) were used as size markers. Sizes are indicated on the left in kilobase pairs. Southern blot (B and C) using DIG‐labeled probes “scaffold 24” and “scaffold 35,” respectively. The probe “scaffold 24” recognized the potential plasmid DNA band observed with the uncut genomic DNA. The intact genomic DNA is recognized by the “scaffold 35” probe, suggesting that this scaffold is a part of the genomic DNA.

**Table S1.** The percentage sequence identity and sequence coverage of the 16S rRNA of *Microvirga massiliensis* with other strains of *Microvirga*.
