# Supplementary figures and images for: Microvirga massiliensis sp. nov., the human commensal with the largest genome
Source: Microbiologyopen. 2016 Jan 8;5(2):307–22. doi: 10.1002/mbo3.329 (PMC4831475; doi:10.1002/mbo3.329)

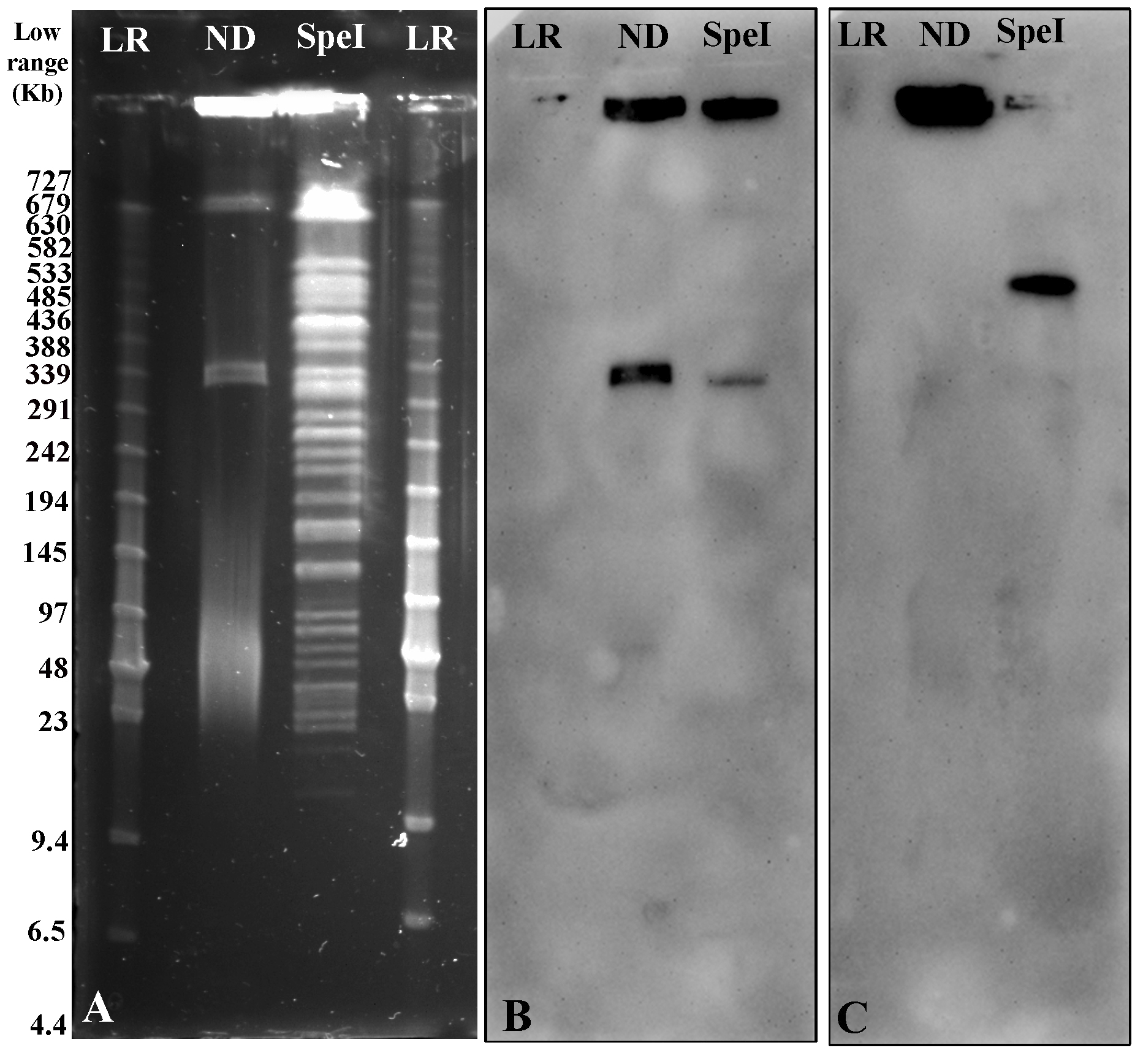

Supplement: Supplementary file 1 — Figure S1. Pulsed field gel electrophoresis (PFGE) and Southern blot (A) PFGE of intact genomic DNA (ND) and SpeI‐digested DNA from Microvirga massiliensis. Electrophoresis was performed in 1% agarose in 0.5× TBE buffer, and the pulse time was ramped from 5 to 50 s for 20 h at a voltage of 5 V/cm for 20 h at 14°C. Gel was stained with ethidium bromide. Low Range PFG Marker (Biolabs, New England) were used as size markers. Sizes are indicated on the left in kilobase pairs. Southern blot (B and C) using DIG‐labeled probes “scaffold 24” and “scaffold 35,” respectively. The probe “scaffold 24” recognized the potential plasmid DNA band observed with the uncut genomic DNA. The intact genomic DNA is recognized by the “scaffold 35” probe, suggesting that this scaffold is a part of the genomic DNA. [file MBO3-5-307-s001.tiff]
