# Supplementary material for: Microvirga massiliensis sp. nov., the human commensal with the largest genome
Source: Microbiologyopen. 2016 Jan 8;5(2):307–22. doi: 10.1002/mbo3.329 (PMC4831475; doi:10.1002/mbo3.329)
Supplement: Supplementary file 2 — Table S1. The percentage sequence identity and sequence coverage of the 16S rRNA of Microvirga massiliensis with other strains of Microvirga. [file MBO3-5-307-s002.docx]

Table S1 The percentage sequence identity and sequence coverage of the 16s rRNA of *Microvirga massiliensis* with other strains of *Microvirga*.
